# INCORPORATING SDOH INTO TRANSITIONAL CARE OF OLDER ADULTS: A THEORETICAL PERSPECTIVE

**DOI:** 10.1093/geroni/igae098.1422

**Published:** 2024-12-31

**Authors:** Onome Osokpo, Karen Hirschman, Elizabeth Shaid, Kathleen McCauley, Mary Naylor

**Affiliations:** University of Illinois Chicago, Chicago, Illinois, United States; University of Pennsylvania, Philadelphia, Pennsylvania, United States; Penn Nursing, Merion Station, Pennsylvania, United States; University of PA School of Nursing, Ardmore, Pennsylvania, United States; University of Pennsylvania, Philadelphia, Pennsylvania, United States

## Abstract

There is solid evidence that Social Determinants of Health (SDoH) contribute to poor patient health outcomes after hospitalization and that transitional care can prevent these adverse outcomes. However, using a standardized strategy for transitional care intervention will result in missed opportunities to enhance positive outcomes for older individuals with chronic illnesses, particularly minority older adults who are greatly affected by adverse SDoH (e.g., food insecurity). Hence, for any transitional care intervention to be effective, it must be adaptable to identify and tackle SDoH issues. In this paper, using the lens of the five domains of the Health People 2023 SDoH framework (Economic Stability, Education Access and Quality, Health Care Access and Quality, Neighborhood and Built Environment, and Social and Community Context), we demonstrate how SDoH can be integrated into an evidence-based transitional care intervention to prevent adverse events such as avoidable emergency room visits and rehospitalization and enhance positive outcomes for patients as they transition from hospital to home or other health care settings. Specifically, we highlight opportunities for the transitional care intervention teams and the health care systems to identify and mitigate SDoH factors using the Transitional Care Model intervention (TCM), a practical and adaptable transitional care intervention, to reduce adverse events and reduce health disparities and health inequities.

